# Current Direction Regulates Ion Transport Across Layer-by-Layer One-Side-Coated Ion-Exchange Membranes in Electrodialysis

**DOI:** 10.1021/acsami.5c00155

**Published:** 2025-03-30

**Authors:** Alaaeldin A. E. Elozeiri, Jouke E. Dykstra, Rob G. H. Lammertink, Huub H. M. Rijnaarts

**Affiliations:** † Environmental Technology, Wageningen University & Research, Bornse Weilanden 9, 6708 WG Wageningen, The Netherlands; ‡ Membrane Science and Technology, Faculty of Science and Technology (TNW), University of Twente, Drienerlolaan 5, 7522 NB Enschede, The Netherlands

**Keywords:** polyelectrolyte multilayer, layer-by-layer, ion transport, bilayer membrane, electrodialysis

## Abstract

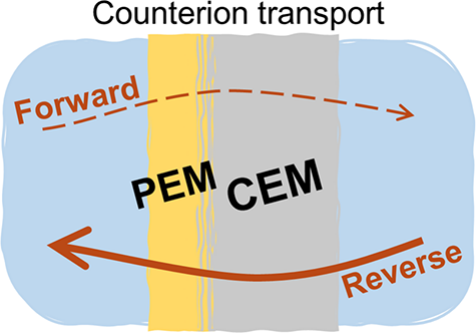

Polyelectrolyte multilayer (PEM) modified membranes can attain selective ion separations in electrodialysis with several potential applications, such as sustainable brine management. To understand the ion transport across PEM-coated membranes, we coated six different commercial cation-exchange membranes (CEMs) with PEM via the layer-by-layer technique. Coating one side of the membrane with a PEM leads to an asymmetric current–voltage response in case of solutions containing Mg^2+^ and Ca^2+^ ions. When the coating faces the counterion transport direction (FT), the coated membrane reaches a limiting current density which does not occur if the applied current is reversed. We investigated these phenomena via several electrochemical techniques. After coating, the total membrane resistance increases significantly at solutions of Mg^2+^ or Ca^2+^ (relative to the bare membrane resistance). Furthermore, the transport characteristics of the PEM coating are highly influenced by the base membrane resistance and fixed-charge density. Regarding the counterion type, the resistance of the coated membrane increases in the same order as the bare membrane: K^+^ < Na^+^< Ca^2+^ < Mg^2+^. The higher the bare membrane resistance is, the higher the PEM resistance is. The co-ion valency (i.e., monovalent Cl^–^ or divalent SO_4_^2–^) had limited to insignificant effects on the current–voltage response of the coated membranes. Therefore, dielectric exclusion is insignificant for these coated membranes at the tested concentrations, i.e., 0.25 M SO_4_^2–^. Lastly, we employed an ion transport model to explain the observed effect of the current direction on the current–voltage response and analyze the effective properties of the PEM coating.

## Introduction

Electrodialysis (ED) and nanofiltration (NF) are membrane-based technologies that are integrated into multiple water treatment applications at various technology readiness levels.^1−3^ While each technology relies on a different membrane type and transport driving force, both technologies can be tuned to support a selective ion separation, e.g., Na^+^/Mg^2+^ separation.^4,5^ Selective ion separations can enhance the circular economy and efficiency of several water treatment and energy storage systems.^6−8^ For example, electrodialysis equipped with monovalent selective ion-exchange membranes was used to separate monovalent ions from divalent ones in seawater brine streams, paving the way toward sustainable brine management practices.^6,9,10^ The ion selectivity can be developed by coating the membrane with a polyelectrolyte multilayer (PEM) via the layer-by-layer technique.^11−13^ In this study, we examine the cation transport across the PEM-modified membranes with a focus on Na^+^, K^+^, Mg^2+^, and Ca^2+^ as they are the main cations in seawater.^2^

In electrodialysis, a potential difference is applied across an ion-exchange membrane (IEM) to drive ion transport.^14,15^ Standard uncoated IEMs provide perm-selectivity where they allow counterion transport and minimize co-ion leakage. Membrane surface modifications can enhance the flux selectivity between different counterions.^16^ The selective coatings can be categorized under four groups: a highly cross-linked layer, an oppositely charged layer, a polyelectrolyte multilayer (or layer-by-layer film), and a dense neutral layer. The influence of the polyelectrolyte multilayer coating conditions on its structure was investigated elsewhere, e.g., the polyelectrolyte type and the ionic strength of the coating solution.^4,11,17,18^

Understanding the transport phenomena across the membrane is key to its technology development and unlocking new water reuse applications. For a coated membrane, the ion transport models considered the coating either as a boundary condition or a separate domain. Saracco and Zanetti modeled the coating of a monovalent-selective IEM as a boundary condition (BC) at the membrane/solution interface.^19^ In this regard, they compared two boundary conditions with different theoretical backgrounds. A thermodynamic BC implies that the coating influences the thermodynamic ion equilibrium between the membrane and the solution. A kinetic BC assumes that the coating imposes a high potential energy barrier to the transport of multivalent ions relative to that of monovalent ions. The thermodynamic BC failed to fit the separation data of a monovalent-selective membrane as it does not describe the effect of the applied current density on the monovalent/divalent separation factor.^19,20^ The kinetic BC provided good fitting for the separation factor of only one of the studied systems, i.e., C_2_O_4_^2–^/Cl^–^ separation (relative error c.a. ± 15%). For SO_4_^2–^/Cl^–^ separation, using a combined approach (thermodynamic + kinetic BC) led to a better fitting (relative error c.a. ± 15%) compared to using only the kinetic BC. As explained later, the three BC approaches cannot describe the current–voltage curve features of the PEM-coated IEM in the present study.

Other theoretical studies regarded the PEM as N alternatingly charged domain(s) next to the IEM domain where N is the number of deposited polyelectrolyte layers.^21−23^ An ionic charge density (ICD) is usually assumed in the layers of the PEM where the charge sign alternates between +1 and −1 depending on the type of the deposited polyelectrolyte. Still, experimental validation is needed for those models. The PEM modeling challenge is a consequence of the experimental difficulty of measuring the PEM properties or the interpretation of the indirect measurements. Furthermore, the properties of each deposited layer of the PEM are even more difficult to measure. While the IEM thickness is on the order of 100 μm,^24^ the PEM coating is on the order of 100 nm.^4,5,11^ The amount of mobile ions inside the PEM is insignificant relative to the mobile ions inside the IEM. Therefore, the direct measurement of the ICD of PEM coating is quite challenging relative to the ICD measurement of the IEM.

Sata et al.^25^ investigated the Na^+^/Ca^2+^ transport through a CEM that is coated on one side/face with a polypyrrole layer. Interestingly, the polypyrrole coating restricted the Ca^2+^ transport only when the coating faced the Ca^2+^ transport direction. Rijnaarts et al.^4^ and White et al.^26^ coated one side of a CEM with a PAH/PSS polyelectrolyte multilayer. On one hand, Rijnaarts et al.^4^ observed selective transport of Na^+^ over Mg^2+^ when the coating faced the counterion transport direction (S^Na^+^ /Mg^2+^^ ∼ 10). However, if the transport direction is reversed, there is no significant increase in selectivity compared to the bare membrane. Contradictorily, White et al.^26^ reported similar K^+^/Mg^2+^ selectivity whether the coating was facing the transport direction (S^K^+^/Mg^2+^^ ∼ 8) or the reverse (S^K^+^ /Mg^2+^^ ∼ 10).

In the present study, we examine the electrochemical features of a one-side-coated ion-exchange membrane and analyze the ion transport via a numerical model. We apply the same coating conditions to different commercial CEMs via the layer-by-layer technique in order to investigate the influence of the bare membrane properties on the coated membrane performance.

## Materials and Methods

Different commercial membranes were investigated: Fujifilm CEM type-10 and type-12 (Fujifilm Manufacturing Europe BV, The Netherlands), Selemion CMTE and CMVN (Asahi Glass Co., Japan), and Fumasep FKS-PET-130 and FKD-PK-75 (Fumatech BWT GmbH, Germany). The bare membrane properties, e.g., membrane thickness, ionic charge density, and resistance, are retrieved from our previous studies.^24,27^ Solutions were prepared using Milli-Q water (Millipore) and the salt(s) of interest (reagent grade): Na_2_SO_4_, KCl, K_2_SO_4_, MgSO_4_, MgCl_2_, and CaCl_2_ (Sigma-Aldrich). Sodium chloride was purchased from VWR Chemicals. Poly(4-styrenesulfonic acid) solution (PSS, ∼ 75 kDa, 18 wt % in H_2_O, 1.11 g/mL at 25 °C) and poly(allylamine hydrochloride) (PAH, ∼ 50 kDa) were purchased from Sigma-Aldrich.

### Layer-by-Layer Coating

**Figure 1:**
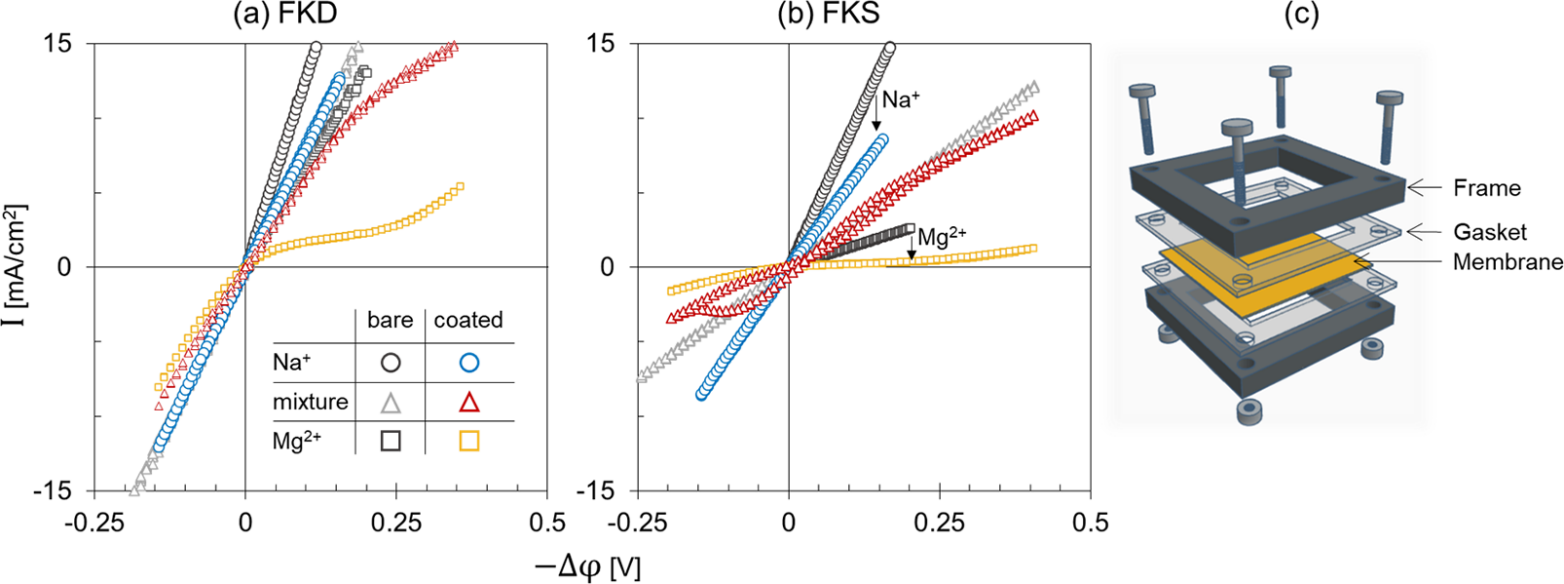
Current–voltage curves of bare and coated membranes of (a) FKD and (b) FKS. The bare membrane data (gray markers) were measured via potential sweep. The coated membrane data (colored markers) are measured via cyclic voltammetry. The coated membranes are cation-exchange membranes with one-side coating of 6.5 PAH/PSS bilayers. The membranes were characterized at three different solutions: 0.5 M NaCl (circles), 0.25 M MgCl_2_ (squares), and a mixture of 0.25 M NaCl + 0.125 M MgCl_2_ (triangles). (c) An illustration of the frame used to hold the membrane during the polyelectrolyte multilayer coating procedure. The membrane is sandwiched between two plastic gaskets to prevent leakage of the coating solution.

Following the recipe of Zhu et al.,^13^ we modified the surface of commercial cation-exchange membranes (CEMs) via poly(allylamine) (PAH)/poly(4-styrenesulfonate) (PSS) layer-by-layer coating. The coating was performed at ambient laboratory temperature (20 °C). Before coating, the membranes were soaked in 0.1 M NaCl overnight. To produce one-side-coated membranes, each membrane sample was mounted horizontally using a homemade holder (Figure 1c) that was 3D printed with acrylonitrile butadiene styrene material (Ultimaker ABS). A solution of polycations or polyanions was gently poured on the top membrane surface and left for 5 min. The polycation solution was prepared as 0.02 M PAH in 1 M NaCl, where we used HCl acid to lower the solution pH from 4.4 to 2.3. The polyanion solution was prepared as 0.02 M PSS in 0.5 M NaCl, where we used NaOH to raise the solution pH from 1.7 to 2.3. First, we deposited the polycation on the cation-exchange membrane as the membrane has anionic fixed-charged groups. After each deposition, the polyelectrolyte solution is removed and the surface is cleaned of weakly adsorbed polyelectrolytes by rinsing both sides of the membrane for 1 min using a squirt bottle of milli-Q water. After the polycation deposition and the rinsing step, the polyanion deposition was performed using a solution of 0.02 M PSS in 0.5 M NaCl (pH 2.3). We deposited 6.5 bilayers (i.e., 13 layers) on 6 different types of CEMs. In addition, 12.5 bilayers (i.e., 25 layers) were deposited on samples of FKD and FKS membranes.

### Electrochemical Characterization

We characterized the electrochemical behavior of the coated membranes via cyclic voltammetry (CV), electrochemical impendence spectroscopy (EIS), and chronopotentiometry (ChP) using a 6-compartment electrodialysis cell (6C-ED, further details are provided in ref (24)). The membrane samples were equilibrated in the solution of interest overnight, where the solution was refreshed three times. First, a blank measurement was made. Then, a membrane sample was inserted between the middle compartments where the solution of interest is circulated. We characterized the membrane samples in different single electrolyte solutions: 0.5 M NaCl and KCl as well as 0.25 M Na_2_SO_4_, K_2_SO_4_, MgSO_4_, MgCl_2_, and CaCl_2_. In addition, the membranes were tested in a binary mixture of 0.25 M NaCl + 0.125 M MgCl_2_. The total equivalent cation concentration in the investigated solutions was kept at 0.5 M to provide a direct comparison between the different experiments. The electrode rinse solution was 0.5 M Na_2_SO_4_. In compartments 2 and 5, the circulated solution was the same as the solution of interest, except for the CaCl_2_ experiment, a solution of 0.5 NaCl was circulated through compartments, 2 and 5, in order to avoid the CaSO_4_ scaling. The distance between the two capillaries is 7.9 ± 0.2 mm when performing measurements without an area reducer. For measurements involving a single electrolyte of Na^+^ or K^+^, the membrane sample was sandwiched between two area reducers whose overall thickness is 4.0 ± 0.1 mm. The active membrane area with and without area reducers are 23.2 ± 0.1 and 4.52 ± 0.04 cm^2^, respectively. Unless otherwise stated, the solutions were recirculated through the 6-compartment cell at 0.27 ± 0.02 L/min and 20 ± 0.5 °C. For EIS, an alternating current (AC) with an amplitude of 10 mA was applied at a frequency range of 10^5^ – 10^–2^ Hz. For cyclic voltammetry, a step of 10 mV was applied. The cyclic voltammetry curves (CVCs) were scanned at 5 or 0.5 mV/s depending on the time needed to approach steady-state values. The measurements were repeated until a stable signal was recorded, especially when applying direct current or voltage over the membranes for the first time after coating.

## Results and Discussion

### Electrochemical Characteristics of PEM-Coated Membranes

We characterized the current–voltage (iV) responses of bare and one-side-coated membranes at different single electrolyte solutions as well as a binary mixture of NaCl and MgCl_2_. At the tested solutions and applied potential, the bare membranes behaved as ohmic resistors whose iV curves are linear (Figures 1a and 1b, additional results are provided in the Supporting Information SI-2). Further insights on the bare membrane conductivity and the ion mobilities are provided in ref (27). Deposition of PAH/PSS polyelectrolyte multilayer (PEM) on one side of a CEM altered the electric response of the membranes. Generally, the current densities supported by the coated membranes are lower (in magnitude) than the current density supported by the corresponding bare membrane for the same applied potential difference and solution (Figures 1a and 1b). At 0.5 M NaCl, the coated membranes exhibited a linear iV curve but with higher resistances relative to the uncoated samples. The coated PEM acted as an additional resistance for the current carried by the Na^+^ ions.

**Figure 2:**
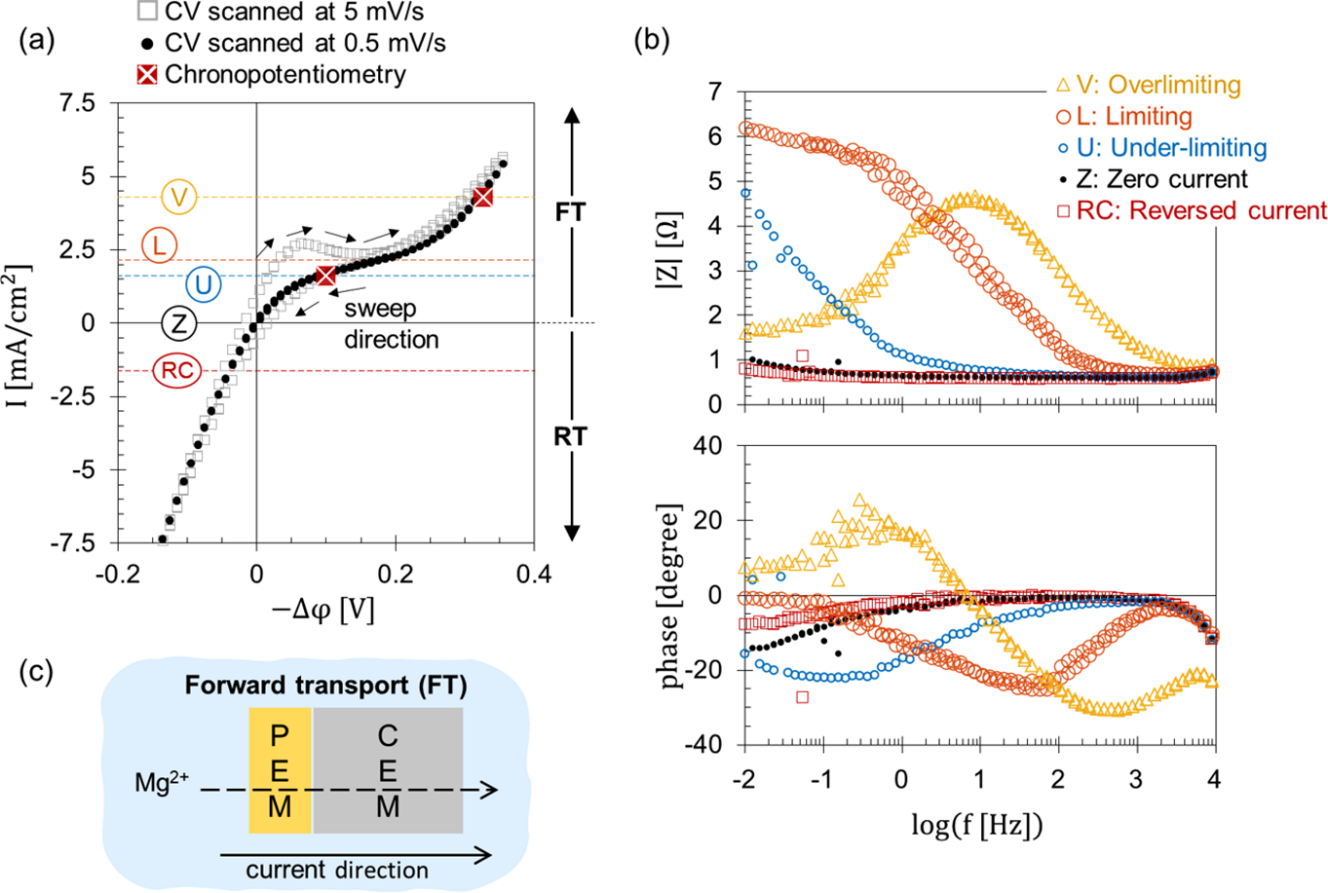
(a) Cyclic voltammetry curve (CVC) of a one-side-coated FKD membrane at 0.25 M MgCl_2_. The cation-exchange membrane (CEM) is coated with 6.5 bilayers of PAH/PSS polyelectrolyte multilayer (PEM). The forward transport direction is indicated by “FT” while “RT” is the reverse transport direction. Chronopotentiometry (ChP) measurements were performed to evaluate the steady-state voltage at certain current densities (marked with an “x” in a red square) (b) The coated membrane was further characterized via electrochemical impendence spectroscopy (EIS). The magnitude and phase of the membrane impedance were analyzed by imposing an alternating current at different frequencies while applying a specific background current density of c.a. −1.6 (reversed current), 0, 1.6 (under-limiting current), 2.2 (limiting current), or 4.3 (overlimiting current) mA/cm^2^. (c) An illustration of the forward transport direction for counterions (FT).

**Figure 3:**
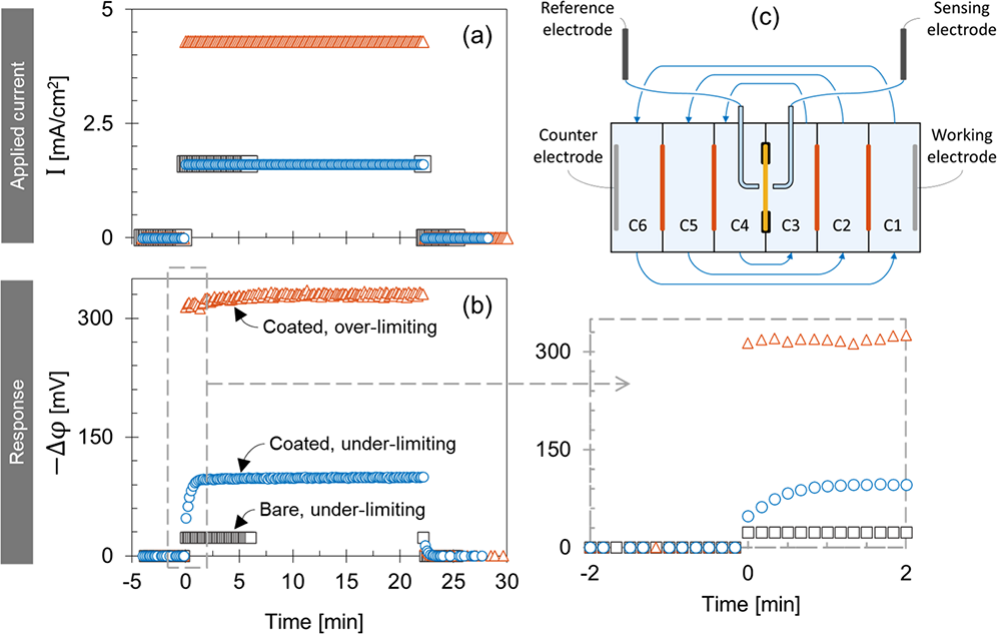
Chronopotentiometric response of bare and coated FKD membrane at 0.25 M MgCl_2_. The membrane was coated with 6.5 bilayers of PAH/PSS. The bare membrane response is presented with gray squares, while the coated membrane is marked by triangles (overlimiting) and circles (under-limiting). (a) The applied current density across the membrane. First, zero current is imposed for 4 min. Then, a constant current is applied for 22 min (coated membrane) or 6 min (bare membrane). Afterward, zero current is imposed again for 2–8 min. (b) The potential drop between the two capillaries of the six-compartment electrodialysis cell (6C-ED). (c) An illustration of the 6C-ED cell where the investigated membrane separates the two middle compartments (C3 and C4). The other compartments are separated by auxiliary ion-exchange membranes. Adapted from Elozeiri et al.^24^ Copyright 2024. Available under a CC BY 4.0 license.

Depending on the scan rate, hysteresis can be recorded in the CVC of the coated CEMs in the presence of Mg^2+^ or Ca^2+^ (Figure 2a). The hysteresis develops when the scanning direction affects the system response at a specific scan rate. By reducing the scan rate, the system response is closer to steady-state values and hysteresis disappears. Moreover, the steady-state response of the membrane can be verified via chronopotentiometry where a constant current is applied for a certain time interval at 0.25 M MgCl_2_ (Figure 3a). The coated FKD membrane (6.5BL) response reached 98% of the steady-state value after 1.7 and 1.8 min in the under-limiting and overlimiting regimes, respectively (Figure 3b). Those durations are quite high when compared to the bare FKD membrane that reached a steady state in 0.2 min. In Section SI-1, we illustrate the progress toward ion equilibria between the solutions and a coated or uncoated membrane under zero current density.

At 0.25 M MgCl_2_, the electric response of the coated membranes depends on the current direction (Figure 2a). When the PEM is facing the transport direction of Mg^2+^ ions (FT: forward transport, Figure 2c), the current does not increase linearly with the applied potential difference. Instead, the current density reaches a plateau, i.e., a limiting current density (Figure 2a). For example, the LCD of the coated FKD membrane is c.a. 2.2 mA/cm^2^, while the uncoated membrane supports a current density of c.a. Twelve mA/cm^2^ at 0.18 V (0.25 M MgCl_2_, Figure 1a). Moreover, overlimiting current densities emerge by raising the magnitude of the applied potential difference (Figure 2a). Different processes can contribute to the overlimiting currents. The overlimiting regime can result from electro-convection vortices as demonstrated for standard and surface-modified IEMs.^28−31^ Besides, the PEM catalyzes the water dissociation into protons and hydroxide ions.^32^ Abdu et al.^17^ and White et al.^26^ recorded significant pH changes in the two solutions separated by a PEM-coated CEM when operating at overlimiting current densities. In most electrolytes, the majority of the current is not carried by protons/hydroxides when applying an overlimiting current through bare membranes, especially for CEMs.^29,33,34^ However, the current carried by the protons across a PEM-coated CEM under an overlimiting current was found as high as ∼ 60% when the terminating layer is the polycation.^17^

We analyzed the coated membrane impedance while applying different direct currents in the background (Figure 2b). The impedance is minimal when a current is applied through the coated membrane in the reverse direction of transport (RT). Moreover, the phase shift is close to 0 at the RT (for frequencies below 10^3^ Hz), which resembles a resistor. At zero net current, the phase shift deviates from zero as the system response is influenced by the changing direction of the applied alternating current. The system impedance increases significantly at under-limiting currents and reaches a plateau at the limiting current density which can result from ion depletion as we explain later. At overlimiting current densities, the system impedance is not monotonic with respect to the frequency. With decreasing the frequency, the system impedance increases to reach a peak, followed by a declining impedance. As explained earlier, the coated membranes take more time to reach a steady state compared to the bare membrane (Figure 3b). Moreover, the decrease in the system impedance at overlimiting (Figure 2b) is a consequence of the production of H^+^ and OH^–^ ions^17,26^ as well as the promotion of ion transport via electroconvection.^28−31^

In our previous work,^24,27^ we calculated the resistance of the bare membranes based on the slope of the current–voltage (iV) curves. Similarly, the resistance of the coated membrane can be calculated in case of single electrolytes of Na^+^ or K^+^ since the iV curve is linear (Figures 1a and 1b, additional results are provided in SI-2). In case of Mg^2+^ or Ca^2+^, the iV curve of the coated membrane is different depending on the current direction. As discussed earlier, the coated membrane behavior approximates an ohmic resistor in the reverse direction (RT, Figure 2a). Therefore, the slope of the RT-direction data points is used to calculate the coated membrane resistance. The membrane area resistance, *R*_m_ [Ω·m^2^], is calculated as follows
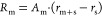
1where *r*_m+s_ [Ω] is the combined (membrane + solution) resistance, *r*_s_ [Ω] is the blank (solution only) resistance, and *A*_m_ [m^2^] is the membrane area. Furthermore, the increase in the membrane area resistance due to the PEM coating, *R*_PEM_ [Ω·m^2^], is formulated as follows
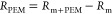
2where *R*_m+PEM_ [Ω·m^2^] is the coated membrane area resistance. Moreover the bare membrane resistivity, ρ_m_ [Ω·m], is calculated as follows:^14^
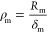
3where δ_m_ [m] is the wet membrane thickness.

**Figure 4:**
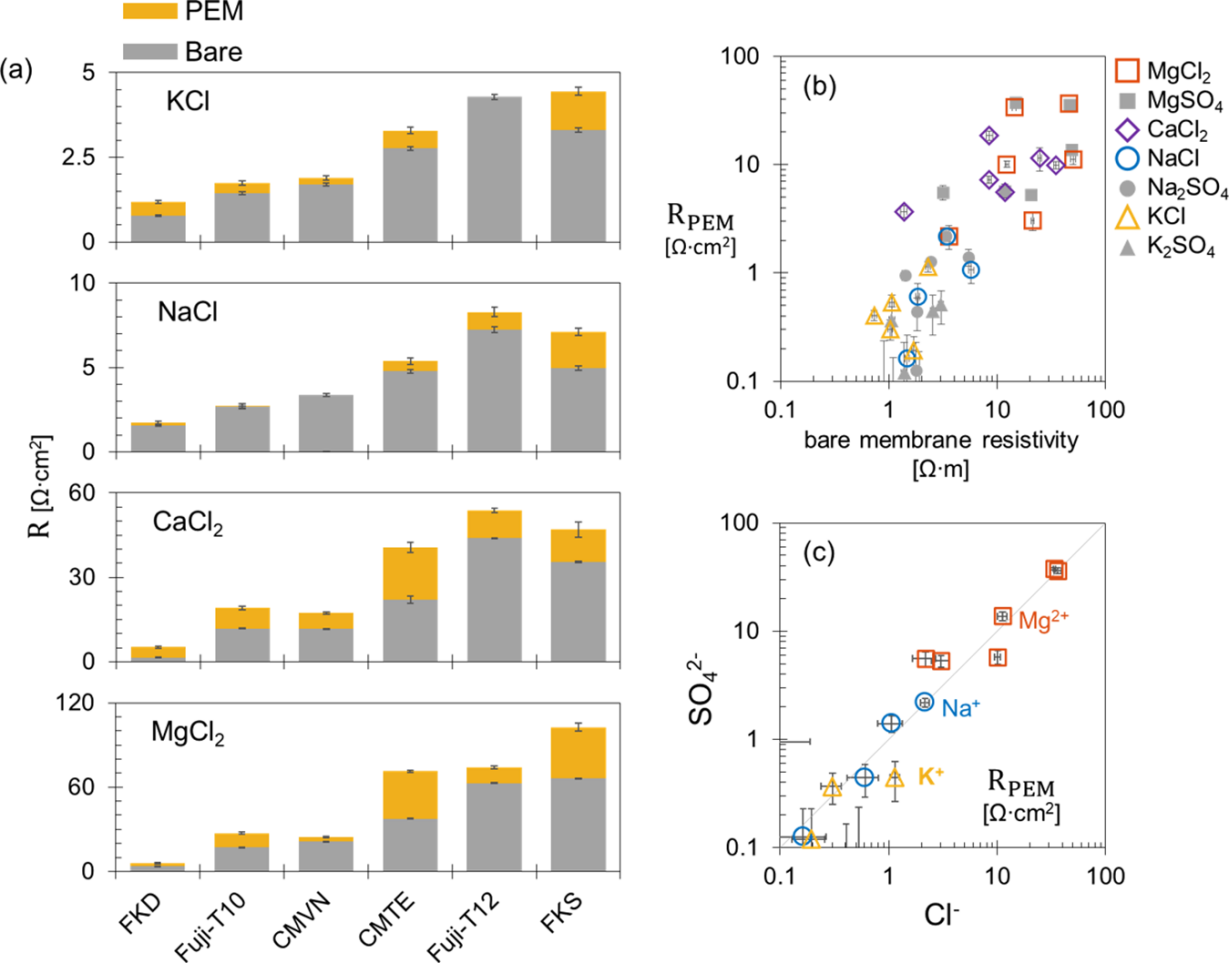
(a) Area resistance of different cation-exchange membranes at single electrolyte solutions of 0.5 M KCl, 0.5 M NaCl, 0.25 M CaCl_2_, and 0.25 M MgCl_2_ based on direct current measurements. The bare membrane resistance is represented by the bottom gray bar. The membranes were coated with 6.5 bilayers of PAH/PSS polyelectrolyte multilayer (PEM) on one side/face. The increase in the total membrane resistance due to the PEM coating is represented by the top yellow bar. The error bar of the PEM resistance is calculated as the summation of the uncertainties in the bare and coated resistances. At Mg^2+^ and Ca^2+^ solutions, the coated membrane resistances are calculated using the current–voltage data points in the ohmic region where the current is applied in the reverse direction (RT). (b) The polyelectrolyte multilayer resistance (*R*_PEM_, *y*-axis) is plotted as a function of the bare membrane resistivity at different single electrolyte solutions (0.5 M KCl/NaCl, 0.25 M K_2_SO_4_/Na_2_SO_4_/CaCl_2_/MgSO_4_/MgCl_2_). (c) The polyelectrolyte multilayer resistance at sulfate solutions (*y*-axis) is plotted versus that at chloride solutions (*x*-axis), e.g., MgSO_4_ vs MgCl_2_. The gray solid line represents the function *y* = *x*.

The resistance of the bare membranes depends on the counterion species.^27^ After coating each membrane with 6.5 PAH/PSS bilayers, the added PEM resistance is of the same order of magnitude as the bare membrane resistance (Figure 4a). The PEM-modified membranes are commonly used for valency-based separations, mainly monovalent/divalent separations.^4,13^ However, significant differences in the coated membrane resistance can still emerge between counterions of the same valency. Generally, the coating resistance increases in the same trend as in bare membranes, i.e., K^+^ < Na^+^ < Ca^2+^ < Mg^2+^. The higher the bare membrane resistivity is, the higher the PEM resistance becomes (Figure 4b). Donnan exclusion can explain that the coated membrane resistance is higher in solutions of divalent cations (i.e., Mg^2+^ and Ca^2+^) compared to solutions of monovalent cations assuming that the PEM coating has a net positive fixed charge density. According to this mechanism, the higher the cation the valency, the lower its concentration becomes inside the PEM which translates to a high resistance. However, Donnan exclusion does not explain the higher coated membrane resistance in MgCl_2_ compared to that in CaCl_2_; since Mg^2+^ and Ca^2+^ carry the same charge. In this regard, ions of higher hydration radius and energy are hypothesized to exhibit a lower mobility inside the PEM coating.

For several nanofiltration (NF) membranes (i.e., PEM-coated ultrafiltration membranes), the salt rejection is relatively high when either the anion, or the cation is a multivalent ion, e.g. the Na_2_SO_4_, MgCl_2_, or MgSO_4_ rejection is high relative to NaCl rejection.^5,11,35,36^ The dielectric exclusion mechanism is usually nominated in literature to explain this rejection trend for NF membranes. The dielectric exclusion mechanism^11^ implies that ions are being rejected due to differences in the dielectric properties between the solutions and the PEM. According to this mechanism, the multivalent ions face higher rejection relative to monovalent ions regardless if they are anions or cations. Tuning the layer-by-layer coating conditions can produce NF membranes with a dominant Donnan exclusion mechanism (over the dielectric mechanism) where the salt rejection depends on the ion valency and charge sign.^5,37^ In the present study, the anion valency (whether monovalent Cl^–^ or divalent SO_4_^2–^) does not have a significant effect on the coated membrane resistances (Figure 4c). Therefore, dielectric exclusion does not have a major role in the ion transport during electrodialysis of the studied concentration, i.e., 0.25 M SO_4_^2–^.

**Figure 5:**
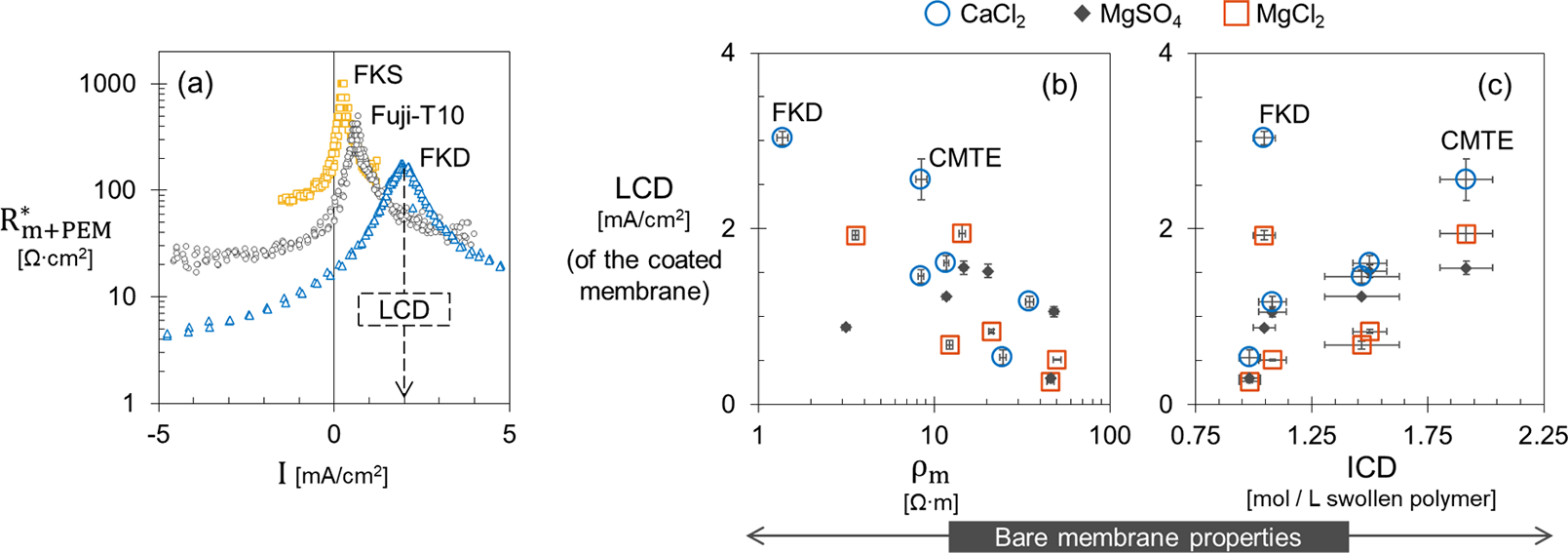
(a) Local effective resistance of three different coated membranes at 0.25 M MgCl_2_ as a function of the applied current density. The limiting current density (LCD) is evaluated at the first local maximum in the positive direction of the applied currents as indicated for FKD membrane by a dashed arrow. The LCD of coated membranes (*y*-axis) versus (b) the bare membrane resistivity (ρ_m_) and (c) the membrane ionic charge density (ICD). The LCD is evaluated at single electrolytes of 0.25 M MgCl_2_, MgSO_4_, and CaCl_2_. Each membrane has one ICD value.

We analyzed the limiting current density (LCD) of the layer-by-layer coated membranes at single electrolytes of 0.25 M MgCl_2_, MgSO_4_, or CaCl_2_. The local effective resistance, *R*_m+PEM_* [Ω·m^2^], is calculated based on the slope of the CVC at each applied current as follows
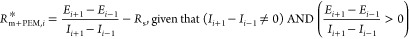
4where *E*_*i*_ [V] and *I*_*i*_ [A/m^2^] are the applied electric potential and current density at point *i* of the CVC, respectively. The local resistance is plotted as a function of the applied current density (Figure 5a). Starting from zero applied current toward positive current values (forward transport direction), the first local maximum is taken as the LCD of the system. At this local maximum, the local resistance of the coated membrane reaches its peak. At higher current densities (after the local maximum), the effective resistance starts to decrease indicating the start of the overlimiting current regime as discussed earlier in Figure 2b.

For single electrolytes containing Mg^2+^ or Ca^2+^, the LCD of the coated membrane is influenced by the bare membrane parameters, namely the bare membrane resistance and the ionic charge density of the membrane (ICD). For MgCl_2_ and CaCl_2_ solutions, the higher the bare membrane resistivity is, the lower the LCD of the PEM-coated membrane (Figure 5b). As we explain later, a high resistivity at the PEM domain can influence both the LCD and the total resistance of the coated membrane.

Except for FKD membrane, coating a high ionic charge density membrane leads to a relatively higher LCD (Figure 5c). The first deposited polyelectrolytes bind to the fixed-charged groups of the bare membrane. The number of binding sites between the polycations and the CEM is hypothesized to affect the charge compensation within the PEM.^38^ The growth of the polyelectrolyte multilayer relies on intrinsic and extrinsic charge compensation.^39,5^ The charged polyelectrolyte can compensate each other via polyelectrolyte complexation, i.e., intrinsic compensation. In addition, mobile ions can compensate for the polyelectrolyte charges, i.e., extrinsic compensation. In this regard, the ionic strength of the polyelectrolyte solution is a critical parameter in the coating procedure. Adsorption of excess polycations via extrinsic compensation leads to net positive fixed charges within the PEM which enhances the Donnan exclusion of the cations and leads to a lower LCD during electrodialysis.

**Figure 6:**
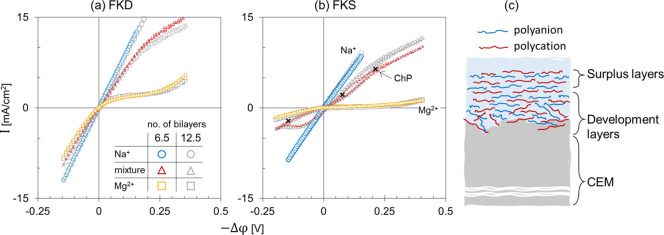
Effect of the number of PSS/PAH bilayers (BL) on the cyclic voltammetry curves of coated (a) FKD and (b) FKS membranes at 0.5 M NaCl (circles), 0.25 M MgCl_2_ (squares), and a mixture of 0.25 M NaCl + 0.125 M MgCl_2_ (triangles). Each membrane was coated with 6.5 and 12.5 bilayers. Chronopotentiometry (ChP) was performed to determine the steady-state voltages at three different applied current densities for FKS-6.5BL at Na^+^/Mg^2+^ mixture. (c) An illustration of the polyelectrolyte multilayer (PEM) structure coated on a cation-exchange membrane (CEM).

We presented a significant influence of the bare membrane properties on the electric response of the coated membranes where all the membranes were coated with the same number of PAH/PSS bilayers (BL). At 0.25 M MgCl_2_, the 6.5BL coated FKS exhibited the highest total resistance (c.a. 100 Ω.cm^2^) while the 6.5BL coated FKD had the lowest resistance (c.a. Six Ω.cm^2^) among the different coated membranes (Figure 4a). We coated new samples of the FKD and the FKS membranes with 12.5 bilayers and characterized their cyclic voltammetry curves (CVCs). Compared to the 6.5BL, there is no significant difference with the CVC of the 12.5BL coated membranes (Figures 6a and 6b). The polyelectrolyte multilayer (PEM) coating can be visualized in terms of two stages: a development and a surplus stage (Figure 6c). The ion transport features of the polyelectrolyte multilayer are shaped during the development zone where the polyelectrolyte deposition is strongly influenced by the base membrane properties. Deposition of additional layers during the development stage has a significant effect on the coated membrane performance as investigated in literature for IEMs^4,17^ as well as NF membranes.^5,35,40^ The type of top-coated layer affects the properties of the PEM/solution interface, e.g., contact angle and surface charge density. Consequently, the top layer influences the water and ion transport, exhibiting an “odd – even” effect.^5,17,37,41^ After the development stage, the additional polyelectrolyte bilayers are considered surplus layers that do not have a significant influence on the electrochemical characteristics of the coated membrane. In other words, the ion transport over the PEM is limited by the development layers.

To test the durability of the electrochemical performance of the PEM-modified membranes, we recorded the cyclic voltammetry curve of FKD membrane coated with 6.5 PAH/PSS bilayers for more than 200 cycles at 0.25 M MgSO_4_ (Figure S5). No significant change in the electrochemical performance of the coated membrane was found over those cycles. This result encourages future efforts to test the long-term stability of the PEM-coated ion-exchange membranes at different temperatures and pH as it will facilitate the commercial application of the PEM-coated IEMs in water treatment processes. In literature, the long-term stability of PEM-modified membranes was investigated for nanofiltration applications. The PAH/PSS coating exhibited a stable performance after exposure to extreme acidic conditions, e.g., 1 M HNO_3_^42^ or 10% H_3_PO_4_ acid solution.^43^ Furthermore, the pH resistance of the PAH/PSS multilayers was enhanced by glutaraldehyde cross-linking.^43,44^ Relative to PAH/PSS, PDADMAC/PSS multilayers showed a higher chemical stability against harsh conditions in case of a hypochlorite solution^45^ or a strong base, e.g., 1 M NaOH.^42^ The PAH/PSS multilayer performance is sensitive to the solution pH as PAH is a weak polyelectrolyte.^42,46^ While PAH protonate at low pH values, it is prone to losing its charge at high pH environment.^47^ Therefore, PAH/PSS coated membranes are recommended to selective cation separations at moderate or acidic pH applications rather than high pH applications.

### Ion Transport Model

**Figure 7:**
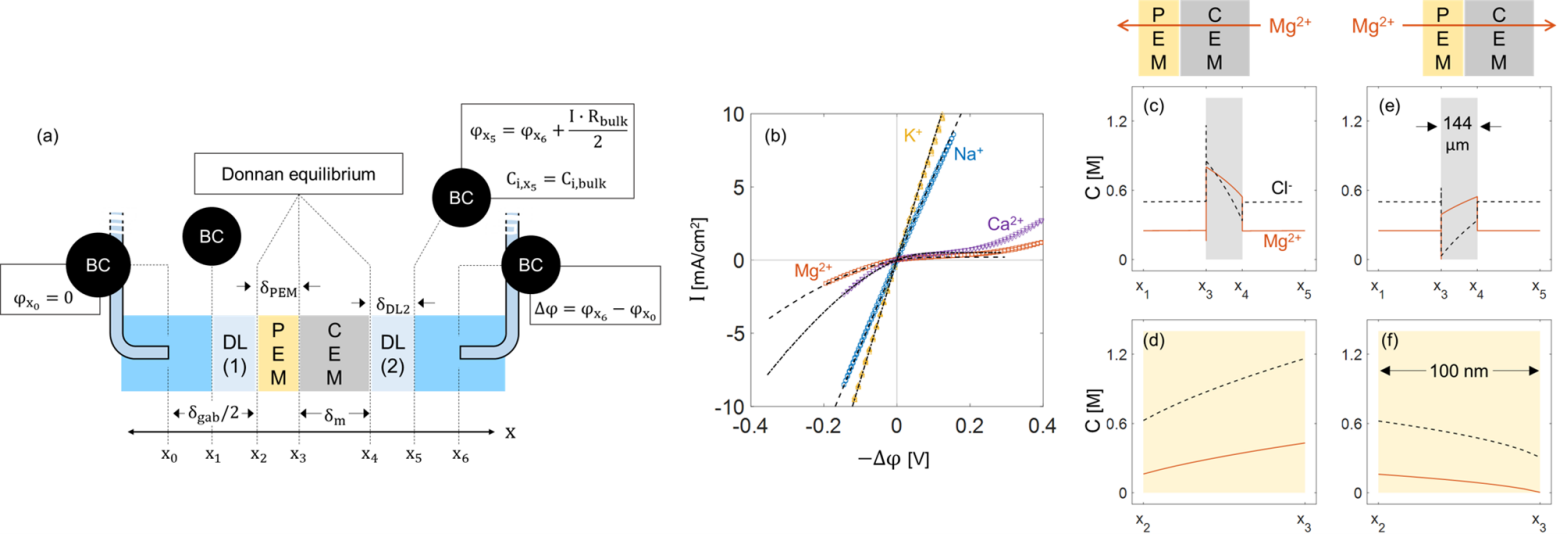
(a) An illustration of 1D ion transport model for polyelectrolyte multilayer (PEM) coated on one side of a cation-exchange membrane (CEM). The coated membrane is placed between two capillaries. The parameters: δ is the layer thickness, C is the ion concentration, ϕ_*x*_ is the electric potential at position *x*, −Δϕ is the applied potential difference, *I* is the applied current density, κ is the conductivity, and *x* is the position across the coated membrane. The abbreviations: “m” is the membrane, “PEM” is the polyelectrolyte multilayer, “CEM” is the cation-exchange membrane, “DL” is the diffusion boundary layer, and “BC” is a boundary condition. (b) Experimental cyclic voltammetry curves (markers) versus the model results (lines) for a coated cation-exchange membrane (CEM) at single electrolyte solutions of 0.5 M KCl (yellow triangles), 0.5 M NaCl (blue circles), 0.25 M CaCl_2_ (downward-pointing triangle), and 0.25 M MgCl_2_ (orange squares). The membrane is FKS membrane which was coated with 6.5 PAH/PSS bilayers on one side via the layer-by-layer technique. The model input parameters are summarized in Table S1. (c and e) Concentration profiles across two diffusion boundary layers and the coated CEM. (d and f) Concentration profiles across the polyelectrolyte multilayer (PEM) coating. The profiles simulate the coated membrane at 0.25 M MgCl_2_ in two cases: (c and d) reverse transport direction where −Δϕ = −0.1 mV and (e and f) forward transport direction where −Δϕ = +0.1 mV.

In this section, we adapted a 1D transport model, from our previous work,^48^ to describe a one-side-coated membrane operated within the under-limiting current regime. The polyelectrolyte multilayer (PEM) is described as an additional domain next to the CEM domain (Figure 7a). A diffusion boundary layer (DL) is considered at each interface of the coated membrane. Donnan equilibrium and electroneutrality conditions are applied at the interfaces between the solution and the coated membrane as well as the interface between the PEM and the CEM. Across the coated membrane and the DL, the ion fluxes are governed by diffusion and migration based on the extended Nernst–Planck equation. Further details are given in SI-3.

Several parameters are required to describe the ion transport through the PEM, including the PEM thickness (δ_PEM_), the ion activity coefficient inside the PEM (γ_i_^PEM^), the ion diffusion coefficient inside the PEM (*D*_i_^PEM^), and the fixed-charge density inside the PEM (*D*_fix_^PEM^) where the subscript, i, refers to a mobile ion. Several studies measured the thickness of a PEM grown over a silicon wafer via ellipsometry in the range of 10 – 700 nm (for 5 – 10 coated bilayers).^4,5,49^ The thickness of the PEM grown on different types of membranes/supports can vary from the measured PEM thickness in the case of a silicon wafer support. In the present study, we aim to describe the electrochemical performance of the PEM-coated CEMs with effective parameters. The PEM resistance depends on the ion diffusion coefficients inside the PEM as well as the PEM thickness. In our model, the PEM thickness is set to 100 nm as a reasonable estimation. Moreover, the ion diffusion coefficients inside the PEM are tuned to describe the PEM resistance.

As discussed earlier, the PEM resistance depends on the bare membrane resistivity (Figure 4b). Therefore, we calculate the mobility reduction factor in the PEM (*r*_F,i_^PEM^) as the ratio between the ion diffusion coefficient inside the bare membrane (*D*_i_^m^) and the ion diffusion coefficient inside the PEM (*D*_i_^PEM^)
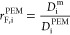
5

Only two fitting parameters are required to describe the current–voltage curve of the coated membrane, i.e., *r*_F,i_^PEM^ and *C*_fix_^PEM^. For single electrolytes, the ion activity coefficients in the solution, PEM, and the membrane are all set to 1. Moreover, a single value was assigned to the mobility reduction factor of the anions and the cations in the PEM.

In Figure S6, we examine the sensitivity of the theoretical iV curve of the membrane to the PEM parameters. The mobility reduction factors inside the PEM (*r*_F,i_^PEM^) affect the system resistance and can be used to capture the current–voltage (iV) curvature at the reverse transport direction (RT, Figure S6a). Moreover, *r*_F,i_^PEM^ influences the limiting current density (LCD) of the coated membrane at the forward counterion transport direction (FT). The higher the mobility reduction factor is, the lower the LCD becomes. The fixed-charge density inside the PEM (*C*_fix_^PEM^) affects the LCD at the forward counterion transport direction (FT) as expected from the Donnan exclusion mechanism (Figure S6b). Using only the *C*_fix_^PEM^ cannot fit the experimental iV curves of the coated membranes. Therefore, we use the *r*_F,i_^PEM^ together with a *C*_fix_^PEM^ to fit the iV curve of the coated membrane.

We fitted the CVCs of the 6.5 BL coated FKS membrane by tuning *C*_fix_^PEM^ to 0.3 M and *r*_F,i_^PEM^ to 1 (KCl), 80 (NaCl), 800 (CaCl_2_) and 1400 (MgCl_2_). Those values describe the coated membrane well in the under-limiting current regime (Figure 7b). To illustrate the influence of the transport direction, we examine the concentration profiles in the CEM and the PEM (Figure 7c–7f). At the forward transport direction (FT), the Mg^2+^ ions move from the PEM domains to the CEM (Figures 7e and 7f). Due to the high resistivity of the Mg^2+^ ions inside the PEM, Mg^2+^ ions get depleted in the PEM at the PEM/CEM interface. As the applied current density is raised, the Mg^2+^ concentration in the PEM decreases at the PEM/CEM interface. At the limiting current density, the Mg^2+^ ions are fully depleted in the PEM at the PEM/CEM interface.

At the reverse transport direction, the Mg^2+^ ions move from the CEM to the PEM. In this case, the Mg^2+^ ions accumulate in the PEM at the PEM/CEM interface which does not restrict ion transport (Figure 7d). Furthermore, the co-ion leakage is higher at the reverse direction where the Cl^–^ ions move from the PEM to the CEM (Figure 7c). As the PEM domain is assigned a net positive fixed charge density, the Cl^–^ concentration is higher in the PEM than in the solution which raises the Cl^–^ concentration in the CEM at the PEM/CEM interface relative to the bare membrane interface with the solution. Therefore, the co-ion leakage is more significant in the reverse transport direction. Rijnaarts et al.^4^ measured the perm-selectivity of similar CEMs at 0.1/0.5 M NaCl where the CEM perm-selectivity decreased after being coated with PAH/PSS multilayers from 92% down to 84 – 87%.

We translated the high PEM resistance toward the multivalent ions into low ion mobilities. The high PEM resistance can also be described by imposing low ion concentrations inside the PEM domain by tuning the ion activity coefficients inside the PEM. Earlier, we summarized the different modeling approaches in literature for the coated membrane. One of the approaches is to account for the selective coating as a modified boundary condition (BC) at the membrane/solution interface^19,20^ rather than another domain. In this regard, Saracco and Zanetti investigated different conditions: a thermodynamic BC (equation 2 in ref (19)) and a kinetic BC (equation 3 in ref (19)). Both BCs do not reflect the electrochemical features of the PEM-coated IEM. In a single electrolyte of MgCl_2_, for example, the current across the coated membrane reaches a limiting value as the potential difference is raised in the forward transport direction (Figures 2a and 2c). Such limiting current density cannot be concluded using the thermodynamic or kinetic BC approach.

## Conclusion

We coated one side of a cation-exchange membrane (CEM) with a PAH/PSS polyelectrolyte multilayer (PEM). The bare membrane resistance increases in the following order of counterions: K^+^ < Na^+^< Ca^2+^ < Mg^2+^. After the PEM coating, the membrane resistance amplifies, while keeping the same order with respect to the counterion species. The low PEM conductivity indicates either low ion mobilities inside the PEM domain, low ion concentrations in the PEM, or a combination of low ion concentrations and mobilities. In case of Mg^2+^ and Ca^2+^, the current–voltage curve exhibits a limiting current density (LCD) when the coating is facing the counterion transport direction. The LCD of the coated membrane is sensitive to the properties of the bare membrane: its resistance and ionic charge density. The effect of the PEM coating on the current–voltage curve resembles a concentration polarization layer where ion depletion occurs at the PEM/CEM interface when the current is in the forward transport direction (FT). In the reversed current direction, ion accumulation takes place. Based on the theoretical model, the PEM resistance influences both the coated membrane (PEM+CEM) resistance as well as the LCD. Furthermore, the fixed-charge density inside the PEM coating influences the LCD while having a negligible effect on the coated membrane resistance.

Cyclic voltammetry (CV) is a reliable tool to compare the performance of the different commercial and novel selective ion-exchange membranes to separate a specific ion. With the aid of chronopotentiometry, the CV scan rate should be tuned to measure the current–voltage response of the membrane at/near steady state. The potential of the coated membranes for a specific ion separation application, e.g., Na^+^/Mg^2+^ separation from a brine stream, can be further examined by measuring the ion flux selectivity in electrodialysis experiments. To find the optimal flux selectivity, the cyclic voltammograms of the coated membranes at single electrolytes can guide the choice of the magnitude and direction of applied current density as well as the base membrane type to be coated.

## Supporting Information Available

Progress toward ion equilibria of coated and bare cation-exchange membranes in contact with a multielectrolyte solution; cyclic voltammetry curves for polyelectrolyte multilayer coated membranes at different electrolytes; 204 cyclic voltammograms of FKD-6.5 PAH/PSS bilayers; and ion transport model for one-side-coated ion-exchange membrane (PDF)
