# Supplementary material for: Current Direction Regulates Ion Transport Across Layer-by-Layer One-Side-Coated Ion-Exchange Membranes in Electrodialysis
Source: ACS Appl Mater Interfaces. 2025 Mar 30;17(14):22004–13. doi: 10.1021/acsami.5c00155 (PMC11986911; doi:10.1021/acsami.5c00155)
Supplement: Supplementary file 1 — am5c00155_si_001.pdf [file am5c00155_si_001.pdf]

Current direction regulates ion transport across layer-by-layer one-side coated ion-exchange membranes in electrodialysis

Alaaeldin A.E. Elozeiri [a], Jouke E. Dykstra [a(*)], Rob G.H. Lammertink [b], Huub H.M. Rijnaarts [a]

[a] Environmental Technology, Wageningen University & Research, Bornse Weilanden 9, 6708 WG, Wageningen, the Netherlands

[b] Membrane Science and Technology, Faculty of Science and Technology (TNW), University of Twente, Drienerlolaan 5, 7522 NB Enschede, The Netherlands

[(*)] Corresponding author. E-mail address: jouke.dykstra@wur.nl

Supporting information (SI)

SI-1. Ion equilibria: coated vs bare membrane

In this section, we examine how the ion concentrations inside the membrane change as the membrane proceeds towards ion equilibria with the solution in contact. We performed this experiment with bare CMVN membranes as well as with layer-by-layer coated membranes. The bare membrane properties are reported in Ref. [1]. For the coated membranes, we coated both sides of CMVN sheets by submerging the membrane in the polyelectrolyte solution of interest (same as reported in the main text). The membrane was coated with 6.5 PAH/PSS bilayers.

Initially, the membranes were equilibrated in a solution of 0.5 M NaCl solution overnight. Afterward, the membranes were submerged in milliQ water in order to desorb excess mobile ions, where the milliQ water was refreshed twice. Afterward, five membrane sheets were stacked in a dialysis stack. The active area of a single membrane sheet is 10 x 4.2 cm^2. The membranes were separated by spacers (thickness: 490 µm). One solution is flowed over through the stack (Figure S1a). Before starting the dialysis experiment, the solution contained 1 mM of each of NaCl, KCl, $MgCl_2$, and $CaCl_2$, while the membrane contained only Na^+ counter-ions. As the solution flowed over the membrane sheets, ions were exchanged between the membrane and the solution. Ion concentrations in the solution were analyzed over time using inductively coupled plasma – optical emission spectrometry (ICP-OES). The concentrations in the membrane (C_i^m) were calculated via a mass balance

$$C_{i,t}^m = C_{i,t-1}^m - \frac{V^s \cdot (C_{i,t}^s - C_{i,t-1}^s)}{V_T^m} \quad \text{Eq. S1}$$

where t refers to the time step, and V^s is the solution volume (i.e., 2 liters). The superscripts, m and s, refer to the solution and the membrane, respectively. The total volume of the stacked membrane sheets (V_T^m) is calculated as follows

$$V_T^m = n \cdot A \cdot \delta_m \qquad \text{Eq. S2}$$

where n is the number of membrane sheets, A is the active area membrane area per sheet, and δ_m is the membrane thickness (i.e., 100 um for CMVN membrane [1]).

For bare membranes, the Na^+ concentration inside the membrane decreases as the Na^+ ions are replaced by the other cations in the system (Figure S1a). The K^+ ions have higher diffusion coefficient relative to Ca^{2+} and Mg^{2+}. Therefore, the K^+ were the fastest to replace the Na^+ ions inside the membrane, where K^+ concentration in the membrane reached its peak in the first 10 min. Afterward, the Mg^{2+} and Ca^{2+} replaced the K^+ and Na^+ inside the membrane. The membrane enabled ion equilibria was achieved within 45 min. The order of counter-ion affinity inside the membrane is: $Na^+ < K^+ < Mg^{2+} < Ca^{2+}$ in agreement with our previous work [2].

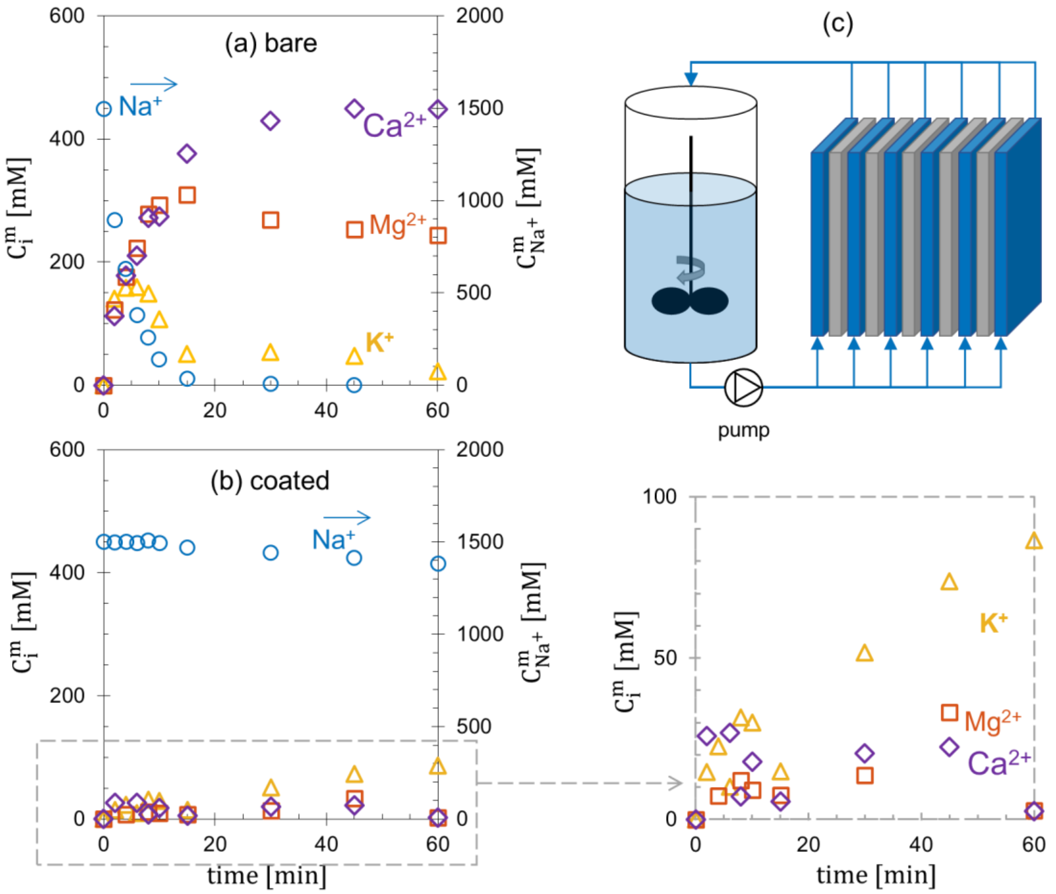

Figure S1. Ion concentrations in (a) bare and (b) coated membrane over time. The K^+ (triangle), Mg^{2+} (square), and Ca^{2+} (diamond) concentrations are plotted via the left y-axis, while the Na^+ concentration (circles) is plotted via the right y-axis. (c) An illustration of the dialysis experiment.

The PEM coating slowed down the exchange between the coated membranes and the solution relative to the case of bare membranes. After 60 min, the counter-ion concentrations inside the membranes did not achieve equilibrium (Figure S1b). Even the K^+ ions exhibited a slow exchange with the Na^+ ions compared to the case of bare membranes. As explained in the main text (Figure 4a), the coated membrane resistance increases for all the cations. Therefore, longer time scales are needed to achieve ion equilibria for PEM-coated membranes compared to the bare ones.

SI-2. Cyclic voltammetry curves

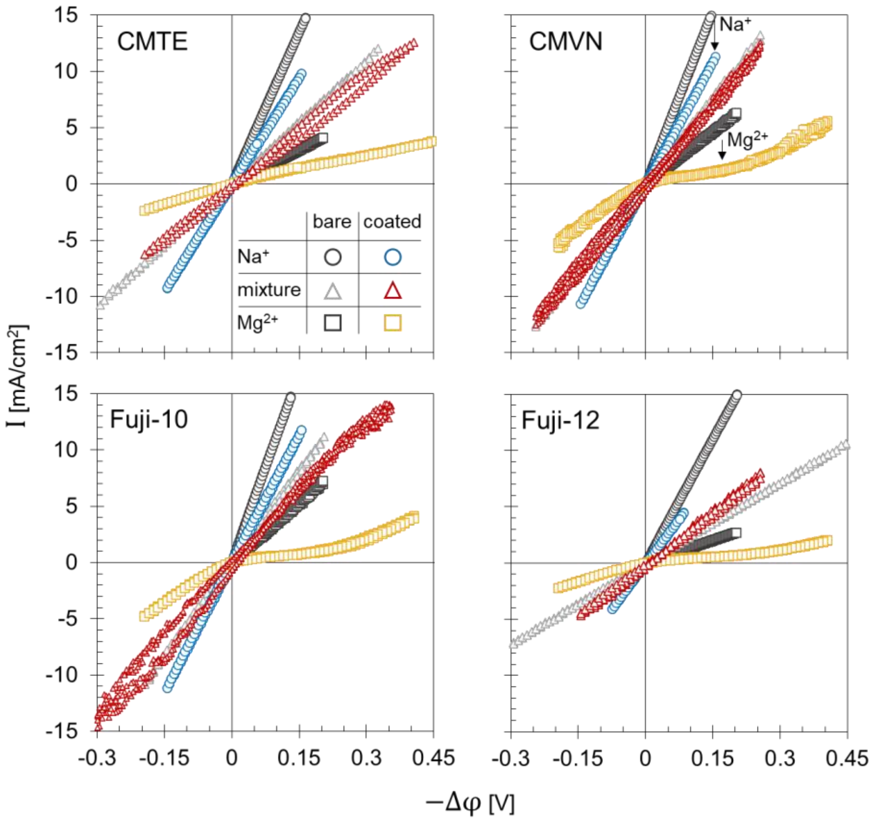

Figure S2. Current-voltage curves of bare and coated membranes of CMTE, CMVN, Fuji-type-10, and Fuji-type-12. The bare membrane data (grey markers) are measured via potential sweep. The coated membrane data (colored markers) are measured via cyclic voltammetry curves (CVCs) after coating one side of the membrane with 6.5 bilayers of PAH/PSS polyelectrolyte multilayer. The membranes were characterized at three different solutions: 0.5 M NaCl (circles), 0.25 M $MgCl_2$ (squares), and a mixture of 0.25 M NaCl + 0.125 M $MgCl_2$ (triangles).

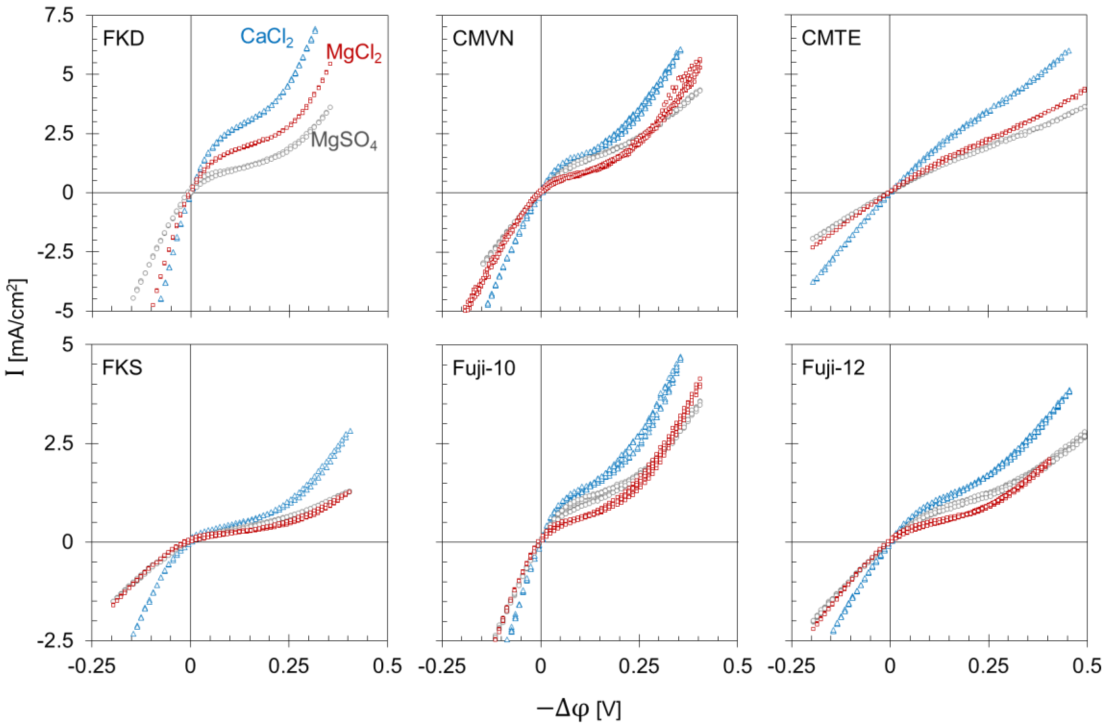

Figure S3. Current-voltage (IV) curves of six different cation-exchange membranes (CEMs) coated with 6.5 PAH/PSS bilayers on one side of the membrane. The investigated CEMs are FKD, CMTE, CMVN, FKS, Fuji-type-10, and Fuji-type-12. The IV curves were measured via cyclic voltammetry and the 6-compartment electrodialysis cell at different single electrolyte solutions: 0.25 M of $MgCl_2$ (red squares), $MgSO_4$ (grey circles), and $CaCl_2$ (blue triangles). The scan rate was 0.5 mV/s and the potential step was 10 mV.

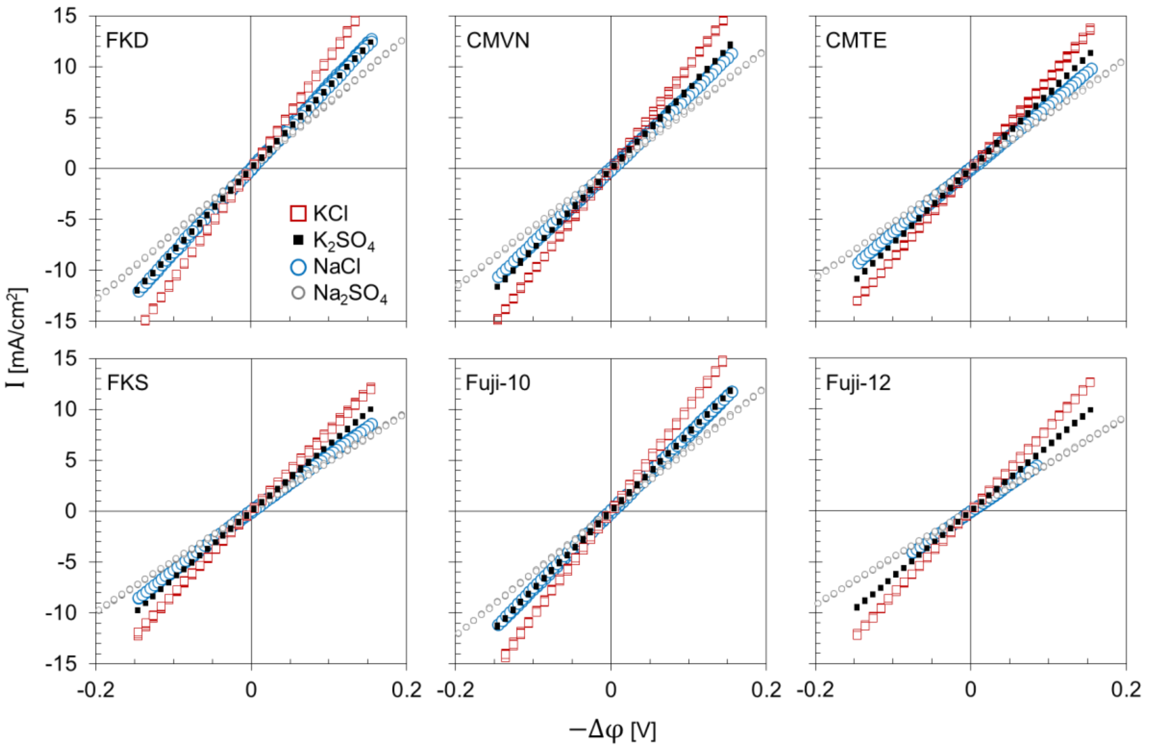

Figure S4. Current-voltage (IV) curves of six different cation-exchange membranes (CEMs) coated with 6.5 PAH/PSS bilayers on one side of the membrane. The investigated CEMs are FKD, CMTE, CMVN, FKS, Fuji-type-10, and Fuji-type-12. The IV curves were measured via cyclic voltammetry and the 6-compartment electrodialysis cell at different single electrolyte solutions: 0.5 M KCl, 0.25 M K_2SO_4, 0.5 M NaCl, and 0.25 M Na_2SO_4. The scan rate was 5 mV/s and the potential step was 10 mV.

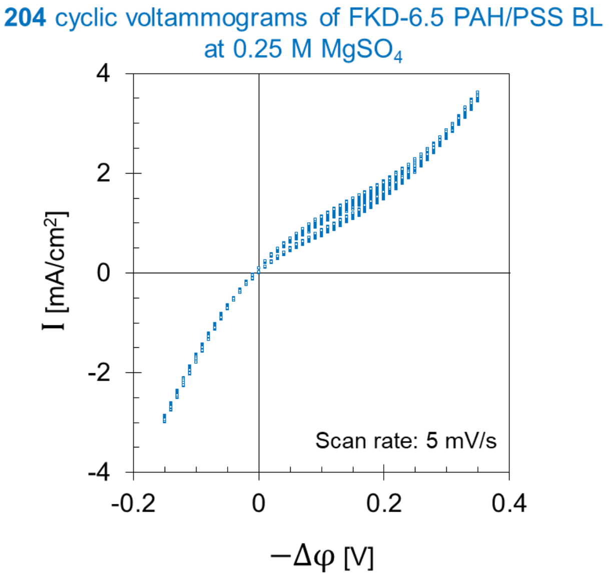

Figure S5. Testing the electrochemical performance durability of the PEM-modified membrane. Cyclic voltammetry was performed on a one-side coated FKD membrane with 6.5 PAH/PSS bilayers (BL) at 0.25 M $MgSO_4$. A total of 204 cycles were recorded at a scan rate of 5 mV/s, a potential step of 10 mV, and solution flow rate of 0.21 L/min.

SI-3. Ion transport model

We employ a 1D model to simulate ion transport across a coated membrane within the under-limiting current regime in a single electrolyte solution, e.g., 0.5 M NaCl (Figure 7 in the main text). The model includes 4 domains: 2 diffusion boundary layers (DL), the polyelectrolyte multilayer (PEM) domain, and the cation-exchange membrane (CEM) domain. We discretize the PEM and CEM domains into 10 elements, each, via the finite difference method [2,3]. The diffusion boundary layer thickness is estimated as 250 µm. As the thickness and the number of discretized elements of the diffusion boundary layer domain do not influence the model results, the DL domains are not discretized, i.e., the DL is composed of 1 element. For simplicity, the activity coefficients in the solution and the membrane are set to 1. The potential in the bulk solution is calculated as follows

$$\varphi^{s}_{x_1} = \varphi^{s}_{x_0} - \frac{I \cdot R_{bulk}}{2} \qquad \text{Eq. S3}$$

where φ is the electric potential, x is the position (as illustrated in Figure 7), I is the current density, and R_{bulk} is the solution resistance (the blank resistance measurement). The superscript, s, refers to the solution. The superscript for the solution is dropped from this point onwards.

The solution/PEM interface is governed by the Donnan equilibrium condition [2] as follows

$$\varphi^{PEM}_{x_2} = \varphi^{s}_{x_2} + \frac{RT}{z_i F} \ln\left(\frac{C^{s}_{i,x_2}}{C^{PEM}_{i,x_2}}\right) \qquad \text{Eq. S4}$$

$$\left(\frac{C^{PEM}_{i,x_2}}{C^{s}_{i,x_2}}\right)^{1/z_i} = \left(\frac{C^{PEM}_{k,x_2}}{C_{k,x_2}}\right)^{1/z_k} \qquad \text{Eq. S5}$$

where R is the universal gas constant, T is the temperature, F is Faraday's constant, z is the ion charge, and C is the concentration. The subscripts, i and k, refer to the ionic species in the system. The superscripts: "PEM" refers to the polyelectrolyte multilayer, and "m" refers to the cation-exchange membrane. Similar to Eq. S4 and S5, Donnan equilibrium condition is applied to the PEM/CEM and the CEM/solution interfaces.

All the model domains are governed by the electroneutrality principle as follows [2]

$$\sum_i z_i \cdot C_i = 0 \qquad \text{Eq. S6}$$

$$z^{m}_{fix} \cdot C^{m}_{fix} + \sum_i z_i \cdot C^{m}_i = 0 \qquad \text{Eq. S7}$$

$$z^{PEM}_{fix} \cdot C^{PEM}_{fix} + \sum_i z_i \cdot C^{PEM}_i = 0 \qquad \text{Eq. S8}$$

where “i” refers to any mobile ion in the system and “fix” refers to the fixed-charged groups, respectively. The current density, I, is calculated as follows [2,4]

$$\mathrm{I} = \mathrm{F}\sum_{\mathrm{i}} \mathrm{z_i} \cdot \mathrm{J_i} \qquad \text{Eq. S9}$$

where the ion fluxes (J) are calculated based on the diffusion and electromigration terms of the Nernst-Planck equation as follows [2,5]

$$\mathrm{J_i} = -\mathrm{D_i}\frac{\mathrm{dC_i}}{\mathrm{dx}} - \mathrm{D_i}\frac{\mathrm{z_i C_i F}}{\mathrm{RT}} \cdot \frac{\mathrm{d\varphi}}{\mathrm{dx}} \qquad \text{Eq. S10}$$

where D_i is the diffusion coefficient of ion, i. As we simulate a steady state, the ion fluxes are constant across all the domains.

Table S1. Model input parameters: δ is the thickness, N_e is the number of discretized elements, D_i is the ion diffusion coefficient in solution, and D_i^m is the ion diffusion coefficient in the membrane based on the direct current resistance measurement. The abbreviation “m” refers to the cation-exchange membrane (CEM), “PEM” refers to the polyelectrolyte multilayer, and “DL” refers to the diffusion boundary layer. (*) those parameters are based on the overall volume of the wet membrane (including the reinforcement material).

Parameter		value		Reference
CEM type		FKS-PET-130		
δ_m		144	[μm]	1
δ_{PEM}		100	[nm]	
δ_{DL1}, δ_{DL2}		250	[μm]	
$N_{e,m}$, $N_{e,PEM}$		10		
$N_{e,DL1}$, $N_{e,DL2}$		1		
C_{fix}^{m} (*)		0.75	[mol/L wet IEM]	1
D_i	Na^+	1.33	[10^{-9} m^2/s]	6
	K^+	1.96		
	Mg^{2+}	0.71		
	Ca^{2+}	0.79		
	Cl^-	2.03		
D_i^m (*)	Na^+	6.07	[10^{-11} m^2/s]	7
	K^+	9.12		
	Mg^{2+}	0.226		
	Ca^{2+}	0.421		

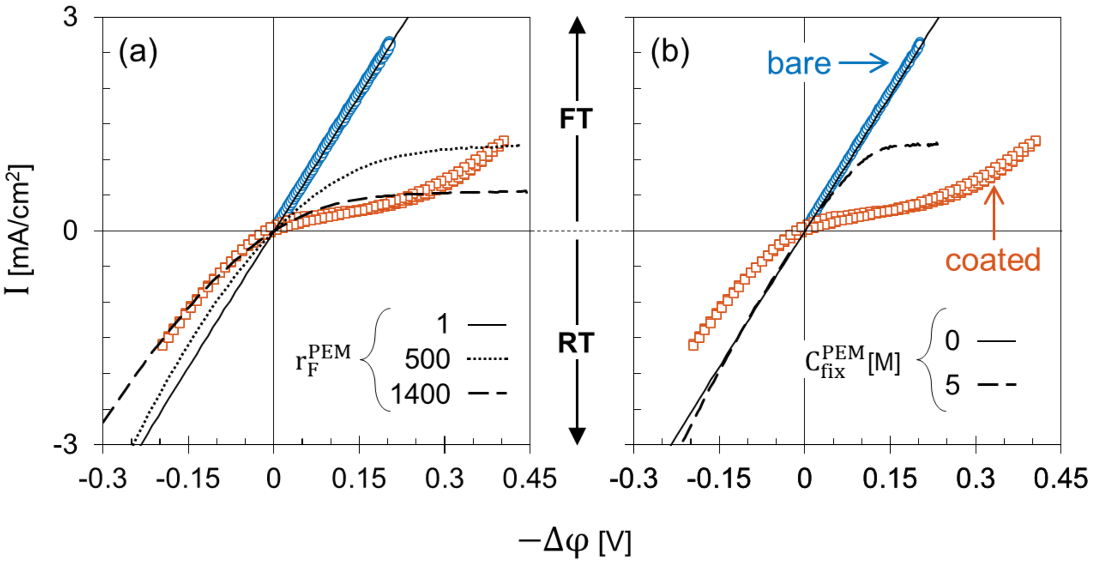

Figure S6. Effect of (a) the mobility reduction factors, $r_{F,i}^{PEM}$, and (b) the fixed-charge density inside the PEM (C_{fix}^{PEM}, on the current-voltage curve at 0.25 M $MgCl_2$. The model results for coated membrane at different parameter values are plotted as lines. The experimental current-voltage values are plotted using blue circles for bare FKS membrane, and using orange squares for a coated FKS membrane with 6.5 PAH/PSS bilayers. (a) Different values of $r_{F,i}^{PEM}$ were tested in the model while keeping all other parameters constant where the C_{fix}^{PEM} was set to 0. (b) Different values of C_{fix}^{PEM} were tested in the model while keeping all other parameters constant where the $r_{F,i}^{PEM}$ was set to 1. The forward transport direction is indicated by "FT" while "RT" is the reverse direction.

References

(1) Elozeiri, A. A. E.; Lammertink, R. G. H.; Rijnaarts, H. H. M.; Dykstra, J. E. Water Content of Ion-Exchange Membranes: Measurement Technique and Influence on the Ion Mobility. *J. Memb. Sci.* **2024**, *698*, 122538. https://doi.org/10.1016/j.memsci.2024.122538.

(2) Elozeiri, A. A. E.; Dykstra, J. E.; Rijnaarts, H. H. M.; Lammertink, R. G. H. Multi-Component Ion Equilibria and Transport in Ion-Exchange Membranes. *J. Colloid Interface Sci.* **2024**, *673*, 971–984. https://doi.org/10.1016/j.jcis.2024.06.025.

(3) Chapra, S. C.; Canale, R. P. *Numerical Methods for Engineers*, 6th ed.; McGraw-Hill: New York, 2010.

(4) Biesheuvel, P. M.; Dykstra, J. E. *Physics of Electrochemical Processes*; 2020.

(5) Strathmann, H. *Ion-Exchange Membrane Separation Processes*, 1st ed.; Membrane Science and Technology, 9; Elsevier Science: Amsterdam, 2004.

(6) *CRC Handbook of Chemistry and Physics : A Ready-Reference Book of Chemical and Physical Data*, 97th ed.; Haynes, W. M., Lide, D. R., Bruno, T. J., Eds.; CRC Press, Taylor & Francis Group: Boca Raton, 2017.

(7) Elozeiri, A. A. E.; Lammertink, R. G. H.; Lin, S.; Rijnaarts, H. H. M.; Dykstra, J. E. Counter-Ion Mobility in Cation-Exchange Membranes: Single Electrolytes versus Mixtures. *J. Memb. Sci.* **2025**, *718*, 123636. https://doi.org/10.1016/j.memsci.2024.123636.
